# Editorial: Puberty: neurologic and physiologic development, volume II

**DOI:** 10.3389/fendo.2025.1596546

**Published:** 2025-03-31

**Authors:** Duarte Henriques-Neto, Miguel Peralta, Adilson Marques

**Affiliations:** ^1^ Research Centre in Sports Sciencces, Health Sciences and Human Development (CIDESD), University of Maia, Maia, Portugal; ^2^ Research Centre in Sports Sciences, Health Sciences and Human Development (CIDESD), University of Trás-os-Montes and Alto Douro, Vila Real, Portugal; ^3^ Interdisciplinary Center for Human Performance, Faculty of Human Motricity, University of Lisbon, Dafundo, Portugal; ^4^ Institute of Environmental Health, Faculty of Medicine, University of Lisbon, Lisbon, Lisbon, Portugal

**Keywords:** central precocious puberty, hypothalamic-pituitary-gonadal, MIR, luteinising hormone, sex hormone-binding globulin (SHBG)

## 1 Introduction

Puberty is the transition from childhood to adulthood, during which secondary sexual characteristics develop and the reproductive system matures. This process is complex and influenced by genetic, hormonal and environmental factors (1). Research suggests that genetics play a significant role, accounting for 50 to 70% of the timing of puberty onset and completion (2).

Genetic regulation of puberty involves a balance between inhibitory and excitatory mechanisms. The inhibitory mechanism is controlled by genes such as *GAD67*, *PDYN*, and *GnIH*, while the excitatory mechanism is influenced by *GnRH*, *GLS*, *KISS1*, and *TTF1* (3). One key player in this process is the melanocortin 4 receptor (MC4R), which belongs to the G protein-coupled receptor family. This receptor regulates energy balance and is linked to both obesity and puberty onset (4). Disruptions in MC4R function can lead to excessive hunger (hyperphagia), alterations in puberty timing, and interactions with the kisspeptin-GnRH system an essential pathway for activating the hypothalamic-pituitary-gonadal (HPG) axis, which drives the development of secondary sexual characteristics (5, 6).

Early activation of the HPG axis leads to the production of gonadotropins, such as luteinising hormone (LH) and follicle-stimulating hormone (FSH), which can result in early puberty, particularly central precocious puberty (CPP) (7). Clinical signs of CPP include thelarche (breast development) or menarche (first menstruation) before age 8 in girls, and the appearance of pubic hair or testicular enlargement before age 9 in boys. These early puberty signs require careful evaluation due to their potential impact on long-term health (7). Given these concerns, there is a growing need to improve CPP diagnosis through clinical assessments, laboratory tests, and imaging techniques. Understanding the mechanisms behind early puberty can help minimise health risks through preventive strategies (8, 9). In this editorial we go through each of the articles published in the Research Topic “*Puberty: Neurologic and Physiologic Development - Volume II*” showcasing the main findings.

## 2 Dimerisation of MC4R may control puberty onset and body size polymorphism

### 2.1 Rational

MC4R in the hypothalamus is linked to the regulation of appetite and energy expenditure. Functional alterations in the MC4R can lead to changes in the timing of pubertal maturation, associated to hyperphagia and leptin resistance.

### 2.2 Findings

In a Xiphophorus fish model MC4R was concluded to be a class A G protein-coupled receptor with physiological and regulatory value, not only important for the Xiphophorus fish but also possibly relevant for understanding metabolic regulation in other fish species and even metabolism and obesity in humans (Liu et al.).

## 3 The value of LH basal values and sex hormone-binding globulin for early diagnosis of rapidly progressive CPP

### 3.1 Rational

The HPG axis is activated by the kisspeptin-GnRH system, which stimulates the production of LH and FSH and the consequent development of secondary sexual characteristics. LH and FSH regulate the synthesis and secretion of oestrogens, progesterone, and the menstrual cycle. A high concentration of LH and FSH at baseline (i.e. LH>0.2IU/L or LH/FSH ratio >0.6) indicate the onset of puberty. In addition, monitoring SHBG can be a biomarker of CPP, as it is a homodimeric peptide that regulates the metabolism of hormones such as oestradiol and testosterone.

### 3.2 Findings

A cohort of 121 girls found that a baseline concentration of LH ≥0.58 IU/L and SHBG ≤58.79 nmol/L can serve as relevant biomarkers for determining rapidly progressive CPP (Zhang et al.).

## 4 Pineal cysts may promote pubertal development in girls with CPP

### 4.1 Rational

CPP can significantly impact young people’s health. The pineal gland is in the epithalamus and is responsible for the synthesis and regulation of melatonin. Pineal cysts are benign intracranial lesions that may or may not cause clinical symptoms.

### 4.2 Findings

In a single-centre study from China it was concluded that pineal cysts correlate with CPP, especially when the cysts are larger than 5 mm (Yang et al.).

## 5 CPP associated with duplicated pituitary

### 5.1 Rational

Dysfunction in the division of the rostral notochord and the prechordal plate during blastogenesis is generally associated with abnormalities in the central nervous system, including pituitary duplication. Nevertheless, pituitary duplication is rare.

### 5.2 Findings

The combination of isolated pituitary duplication and CPP is rare in clinical contexts. Its prognosis is limited, and meticulous and continuous follow-up is suggested to check for temporal changes. Even so, this clinical case with a literature review shows that treatment with inhibitors seems to have therapeutic efficacy (Yang et al.).

## 6 Development of CPP in a girl with late-diagnosed simple virilising congenital adrenal hyperplasia complicated with Williams syndrome

### 6.1 Rational

Willams syndrome is a genetic disorder characterised by delayed development, mental disorders, and abnormalities in the cardiovascular and renal systems.

### 6.2 Findings

This is a case study of a 4-year-old girl with Williams Syndrome and congenital adrenal hyperplasia (CAH) who developed CPP after starting corticosteroid treatment. The patient showed progressive virilisation, developmental delay and advancing bone age. Genetic tests confirmed mutations in the *CYP21A2* gene related to CAH. However, to mitigate underdiagnosis and improve prognostic outcomes, it is imperative to consider concomitant disorders that present multiple atypical clinical features (Joo et al.).

## 7 Magnetic resonance imaging for assessing cranial pathologies in rapidly progressive early puberty cases

### 7.1 Rational

CPP can lead to organic pathologies in the central nervous system, especially if progressing rapidly. One way of assessing these changes at the cranial level is through MRI.

### 7.2 Findings

This research highlights the need for MRI imaging for children between 6-8 years of age continues to be debated, the results suggest that imaging decisions should be individualised (Kılınç Uğurlu et al.).

## 8 Conclusion

Adolescence is characterised by important physiological and psychological changes that impact present and future health. This editorial highlights the importance of developing in-depth knowledge of pubertal processes and the need for methodologies and tools for diagnosing CPP. The role of the MC4R in regulating the onset of puberty and body weight was highlighted by its differentiated dimerisation. The baseline values of LH and SHBG proved useful for the early diagnosis of rapidly progressing CPP, allowing for more timely interventions. MRI has also revealed that some intracranial abnormalities are associated with CPP, although in very specific cases, such as the presence of pineal cysts.
